# Case, No. 11 and 12

**Published:** 1863-07

**Authors:** 


### Editorial

### A CASE—No. 11.

ABOUT two years ago we filled several teeth for Mrs. B., among which was a second superior left bicuspid, very much decayed; the crown about two-thirds gone, and the pulp dead. There had not at any time been periostitus about it. The proper excavation was made, the canal cleaned out and filled; the crown was brought up to an approximation of the original form, by the filling. About three months after the tooth was filled, it was suddenly and severely attacked by periostitus, and gave intense pain, during one night and the next day till the afternoon, when menstration occurred. At this juncture the pain subsided, and the soreness, swelling and inflammation left the parts about as rapidly as they had occurred. Since that time there has not been the slightest inflammation or soreness about the tooth. No abscess was formed, no treatment was employed. The lady was about twenty-eight years old, of nervous sanguine temperament, the former largely predominating.

There was also filled about the same time, a first right inferior bicuspid, largely decayed on the posterior proximal surface; its pulp was also dead. Filled both fang and decayed cavities.

About three weeks ago, menstration occurred as usual; the next day after its occurrence, Mrs. B. was exposed by being out shortly after a rain; that evening the flow was entirely arrested, when this inferior bicuspid became suddenly and intensely painful,—active periostitus occurred immediately after the suppression. This continued occasioning almost an incessant agony for several hours, when we were called to the case.

She had slept little or none during the night; the parts were somewhat swollen, and increasing in this particular. She had been using tincture of iodine upon the gums for several hours before we saw it, without any perceptible effect. We immediately applied a leech to the gum,—the bleeding continued for about three hours after the leech was removed. This gave but little relief, and another night of severe pain was passed. After the leeching the patient was directed to use a warm foot bath, with a view of equalizing the circulation, and with a hope of inducing a return of the menstral flow; it did not have the desired effect, at that time at least. Next morning about 9 o'clock, there was a slight return of the menses; the pain ceased, and the soreness and swelling began to abate immediately, and in a short time was entirely removed. The point most prominent in these cases is the influence of menstration upon them, as here exhibited in two directions.

There are doubtless many cases subject to such influences that escape observation altogether.

During the period of gestation, feeble teeth are subject to great vicissitudes. Observations and accurate reports upon these subjects would be interesting and important. T.

---

A CASE—ITS TREATMENT—No. 12.

July 8th. Mrs. P. aged 25 years, of sanguine lymphatic temperament and of good constitution, called for consultation in regard to the right lateral superior incisor. The tooth was much decayed on mesial-proximal surface: the pulp had become exposed some months previous. Before this, however, the tooth had been twice filled by some one,—the filling dropped out each time. An application of arsenic was made by the same dentist, (?) to destroy the pulp, which was only partially done; the vitality was gone about half way up the fang: the remaining portion retained its vitality and sensitiveness in full, at least so it seemed. There had been periostitus all the time since the application, so much so, that the tooth was very sore to the touch; the inflammation had assumed a chronic character: there was no abscess.

Treatment.—Removed the remaining portion of the pulp, accomplished this by an operation; thought arsenic strongly counter indicated. From the moment of the removal of the pulp the soreness diminished, the depletion perhaps was the occasion of this. Then scarified the gum and applied tincture of iodine, to induce counter-irritation; this made some improvement, but not to the desired extent: then applied a leech to the gum and continued the tincture of iodine. Used creosote in the fang from the time of the removal of the pulp. This treatment occupied three days, at the end of which time the periostitus was much reduced. Then introduced a few fibers of prepared flax moistened with creosote, into the canal of the fang near its apex, then proceeded to fill the fang and decayed cavity firmly with gold, using the mallet, the first few strokes of which occasioned some pain; this became less and less as the operation progressed, till at its completion there was a very slight soreness remaining. Periostitus is very seldom an obstacle to the use of the mallet.

